# Exploring the role of eosinophil cationic protein (ECP) in schizophrenia: Insights and implications

**DOI:** 10.1097/MD.0000000000038380

**Published:** 2024-05-31

**Authors:** Emmanuel Ifeanyi Obeagu

**Affiliations:** a Department of Medical Laboratory Science, Kampala International University, Uganda.

**Keywords:** biomarkers, ECP, eosinophil cationic protein, immune dysregulation, neuroinflammation, schizophrenia, therapeutic targets

## Abstract

Schizophrenia, a multifaceted neuropsychiatric disorder characterized by disruptions in perception, cognition, and behavior, has been associated with neuroinflammatory processes. Emerging research has increasingly recognized the potential involvement of immune-related factors in the pathogenesis of schizophrenia, prompting investigations into biomarkers associated with inflammatory cascades. Among these biomarkers, Eosinophil Cationic Protein (ECP), traditionally known for its role in eosinophil-mediated immune responses, has garnered attention for its putative association with neuroinflammation in schizophrenia. This paper critically examines the current understanding of the role of ECP in schizophrenia. ECP, a cytotoxic protein released by eosinophils, has diverse immunomodulatory effects and has been identified in altered concentrations in individuals with schizophrenia. Studies have reported elevated levels of ECP in peripheral fluids of schizophrenia patients, suggesting a possible link between ECP dysregulation and the inflammatory milieu characteristic of the disorder. Moreover, the potential implications of ECP in neuroinflammatory processes relevant to schizophrenia pathophysiology are discussed. ECP’s role in modulating immune responses and its potential impact on neuronal function, synaptic plasticity, and neurotoxicity within the central nervous system (CNS) are considered, highlighting the potential contribution of ECP to the neuroinflammatory mechanisms underlying schizophrenia. In conclusion, while the precise role of ECP in schizophrenia pathogenesis warrants further elucidation, exploring its association with neuroinflammation holds promise in unraveling new biomarkers and therapeutic avenues for managing this complex psychiatric disorder.

## 1. Introduction

Schizophrenia stands as a multifaceted neuropsychiatric disorder characterized by disturbances in perception, cognition, emotions, and behavior. The etiology of schizophrenia remains complex, involving intricate interactions among genetic, environmental, and neurobiological factors. Recent advances in psychiatric research have unveiled a growing interest in the role of inflammation and immune dysregulation in the pathophysiology of schizophrenia, offering a novel perspective beyond the conventional neurochemical hypotheses.^[1–4]^ In this context, exploring immune-related biomarkers has emerged as a promising avenue to elucidate the underlying mechanisms contributing to the inflammatory cascade observed in schizophrenia. Among these biomarkers, eosinophil cationic protein (ECP), traditionally recognized for its involvement in eosinophil-mediated immune responses, has gained attention as a potential player in the neuroinflammatory pathways implicated in schizophrenia.^[5]^ ECP, a cytotoxic protein predominantly released by eosinophils upon activation, exhibits antimicrobial properties and regulatory effects on immune cells. Beyond its classical role in host defense against parasites and allergic responses, recent studies have begun to explore its broader immunomodulatory functions, prompting investigations into its potential involvement in neuroinflammatory processes relevant to schizophrenia.^[6]^

Notably, investigations examining peripheral biomarkers in schizophrenia have reported alterations in ECP levels in individuals with the disorder.^[7]^ These observations have sparked interest in understanding the relationship between ECP dysregulation and the inflammatory milieu characterizing schizophrenia. However, the specificity and mechanistic implications of ECP alterations in schizophrenia pathogenesis necessitate further scrutiny. The potential role of ECP in neuroinflammatory cascades relevant to schizophrenia remains intriguing.^[8]^ ECP’s interactions within the central nervous system (CNS) and its potential impact on neuronal function, synaptic plasticity, and neurotoxicity present intriguing prospects. Understanding how ECP-mediated immune responses influence the delicate balance of neuroinflammatory processes in schizophrenia could offer valuable insights into the disorder’s underlying pathophysiology.

This paper aims to consolidate and critically evaluate current literature pertaining to the potential role of ECP in schizophrenia. Exploring its association with immune dysregulation and neuroinflammation provides a platform for discussing its putative role as a biomarker and its implications for novel therapeutic strategies targeting the inflammatory pathways in schizophrenia.

## 2. Eosinophils

Eosinophils, a subtype of White blood cells traditionally associated with allergic responses and parasitic infections, have garnered attention beyond their canonical roles due to their potential implication in neuroinflammatory conditions such as schizophrenia.^[9]^ Although historically considered primarily peripheral cells, recent studies have indicated their presence within the CNS, suggesting their possible involvement in neuroinflammatory processes relevant to schizophrenia. Peripheral eosinophil counts have been observed to fluctuate in individuals with schizophrenia. Studies have reported altered eosinophil levels in the peripheral blood of schizophrenia patients, although discrepancies exist among findings.^[10]^ These observations have sparked interest in understanding the systemic immune alterations present in schizophrenia and their potential implications for CNS inflammation.

While the primary function of eosinophils within the CNS remains debated, mounting evidence suggests their infiltration into the brain under pathological conditions. postmortem studies and experimental models have detected eosinophils in CNS tissues, raising questions about their possible roles in neuroinflammatory responses and their contribution to the pathophysiology of schizophrenia.^[11]^ Eosinophils release various factors, including ECP, major basic protein, and eosinophil-derived neurotoxin, which have cytotoxic effects and modulate immune responses.^[11]^ These eosinophil-derived proteins may potentially influence neuroinflammation and neuronal function, contributing to the complex inflammatory milieu characteristic of schizophrenia. Eosinophils have been implicated in interactions with the blood-brain barrier (BBB), which serves as a protective interface between the CNS and systemic circulation.^[12]^ Dysregulation of eosinophil-BBB interactions might lead to compromised barrier integrity, facilitating the infiltration of immune cells and inflammatory mediators into the CNS, thereby impacting neuroimmune crosstalk relevant to schizophrenia pathogenesis. The potential influence of eosinophil-derived factors on neural circuits and neurotransmitter systems could contribute to the development or exacerbation of psychiatric symptoms in schizophrenia. Eosinophil-mediated immune dysregulation might further exacerbate neuroinflammation, potentially impacting neuronal function and synaptic plasticity, thereby influencing disease progression and symptom severity in schizophrenia.

## 3. Eosinophil cationic protein

ECP, a cytotoxic protein predominantly released by eosinophils, has gained attention in the context of schizophrenia due to its potential involvement in neuroinflammatory processes within the CNS.^[13]^ ECP, known for its antimicrobial properties and modulatory effects on immune responses, has been explored in various inflammatory conditions and is emerging as a putative contributor to neuroinflammation in schizophrenia. Study investigating peripheral biomarkers in schizophrenia has reported alterations in ECP levels in blood or cerebrospinal fluid of affected individuals.^[14]^ Elevated ECP levels have been observed in some cases, suggesting a potential association between ECP dysregulation and the neuroinflammatory milieu characterizing schizophrenia. However, discrepancies among study findings necessitate further exploration to ascertain the specificity and reliability of ECP alterations in schizophrenia.^[15]^

ECP’s presence within the CNS and its potential impact on neuroinflammatory mechanisms in schizophrenia are of interest.^[8]^ ECP, upon release, can exert cytotoxic effects on various cells and modulate immune responses. Its ability to interact with neuronal cells or glia and its potential influence on neuronal function and synaptic plasticity suggest a plausible role in the neuroinflammatory cascade observed in schizophrenia. ECP’s cytotoxic properties, including its ability to induce cell membrane damage and trigger apoptosis, raise questions about its potential neurotoxic effects within the CNS.^[16]^ ECP’s interactions with neural cells might disrupt synaptic integrity, neuronal signaling pathways, or contribute to oxidative stress, potentially impacting neural function and neuronal viability relevant to schizophrenia pathology.

Understanding the mechanistic involvement of ECP in neuroinflammation associated with schizophrenia offers potential therapeutic implications. Strategies targeting ECP-related pathways or modulating its cytotoxic effects might represent novel avenues for interventions aimed at mitigating neuroinflammatory processes and ameliorating disease progression in schizophrenia. Despite emerging evidence, several challenges remain in elucidating the precise role of ECP in schizophrenia pathophysiology. Clarifying the dynamics of ECP alterations, its specific impact on CNS cells, and its contribution to the overall neuroinflammatory milieu in schizophrenia requires further detailed investigation.

## 4. Various assays to measure eosinophil cationic protein, for the importance of standardization of this parameter

Measuring ECP levels is crucial for understanding its role in various conditions, including allergic diseases, asthma, and potentially schizophrenia. Standardization of ECP measurement is essential to ensure consistency, reliability, and comparability of results across different laboratories and studies. Several assays are used to measure ECP, and standardization becomes particularly important for accurate interpretation and meaningful comparisons.^[16]^ Here are some commonly used assays:

1. **Radioimmunoassay:**
  ○ **Principle:** Utilizes a radioactive-labeled antibody to measure ECP levels.
  ○ **Advantages:** Sensitive and specific.
  ○ **Importance of Standardization:** Standardization is crucial for ensuring uniformity in antibody binding and signal detection across different laboratories.
2. **Enzyme-Linked Immunosorbent Assay:**
  ○ **Principle:** Utilizes an enzyme-labeled antibody to measure ECP levels.
  ○ **Advantages:** High sensitivity and specificity, widely used.
  ○ **Importance of Standardization:** Standardization is necessary to minimize variations in antibody binding, reagent concentrations, and detection methods.
3. **Chemiluminescent Immunoassay:**
  ○ **Principle:** Measures the light emitted during a chemical reaction involving enzymes and substrate.
  ○ **Advantages:** High sensitivity, broad dynamic range.
  ○ **Importance of Standardization:** Ensures consistency in reagent concentrations, incubation times, and detection systems.
4. **Fluorescent Immunoassay:**
  ○ **Principle:** Uses fluorescence-labeled antibodies to measure ECP levels.
  ○ **Advantages:** High sensitivity, allows for multiplexing.
  ○ **Importance of Standardization:** Standardization is essential for consistent fluorescence signals and accurate quantification.
5. **Flow Cytometry:**
  ○ **Principle:** Measures the fluorescence of individual cells, allowing for the quantification of ECP in eosinophils.
  ○ **Advantages:** Provides information on cell-specific ECP levels.
  ○ **Importance of Standardization:** Standardization is necessary for consistent sample preparation, instrument settings, and data analysis.
6. **Western Blotting:**
  ○ **Principle:** Separates proteins based on size and uses antibodies to detect ECP.
  ○ **Advantages:** Allows visualization of ECP protein bands.
  ○ **Importance of Standardization:** Standardization is crucial for reproducibility in protein separation, transfer, and antibody binding.
7. **Immunohistochemistry:**
  ○ **Principle:** Uses antibodies to visualize ECP in tissue sections.
  ○ **Advantages:** Allows for the localization of ECP within tissues.
  ○ **Importance of Standardization:** Standardization is essential for consistent staining protocols, antibody concentrations, and image analysis.

## 5. Eosinophil cationic protein in immune responses

ECP, a cytotoxic protein released by eosinophils, plays a crucial role in immune responses, particularly in host defense against pathogens and modulating inflammatory reactions. ECP’s multifaceted functions contribute to various facets of immune regulation, impacting both innate and adaptive immune mechanisms.^[16]^ ECP exhibits antimicrobial properties by disrupting microbial cell membranes, making it effective against bacteria, viruses, fungi, and parasites.^[17]^ Its cationic nature allows interaction with negatively charged microbial surfaces, leading to membrane permeabilization and subsequent microbe destruction, thereby contributing to the innate immune defense against invading pathogens.

Beyond its direct antimicrobial activity, ECP exerts immunomodulatory effects by influencing immune cells and cytokine production. It can interact with various immune cells, including T cells, B cells, dendritic cells, and mast cells, altering their function and cytokine release. ECP can promote a shift in the immune response by modulating the balance between Th1 and Th2 cytokine profiles, impacting the adaptive immune system.^[17]^ During immune responses, elevated levels of ECP are often observed in inflamed tissues. ECP’s cytotoxic effects, including its ability to induce apoptosis and promote inflammation by stimulating the release of pro-inflammatory cytokines, contribute to tissue damage at the site of inflammation. While crucial for combating pathogens, excessive ECP release can exacerbate tissue injury and inflammation in certain conditions.^[16]^

In allergic reactions, ECP participates in the immune response against allergens. It is involved in the pathogenesis of allergic diseases, such as asthma and allergic rhinitis, by contributing to tissue damage and inflammation in response to allergen exposure. ECP’s presence in allergic conditions reflects its role in the amplification of inflammatory responses.^[18,19]^ Recent studies have explored ECP’s involvement in nontraditional roles, such as its potential implication in neuroinflammation and neuropsychiatric disorders like schizophrenia. While its role in these contexts remains under investigation, the emerging evidence suggests that ECP might influence neuroinflammatory processes and neuronal function, hinting at broader implications in conditions beyond its conventional roles.

## 6. Eosinophil cationic protein as a biomarker in schizophrenia

ECP has emerged as a potential biomarker in schizophrenia, contributing to the evolving understanding of immune dysregulation in this complex neuropsychiatric disorder. While primarily known for its role in eosinophil-mediated immune responses, recent investigations have explored its association with the inflammatory milieu observed in schizophrenia, offering insights into its potential utility as a biomarker for disease monitoring and characterization.^[20,21]^ Studies examining peripheral biomarkers in schizophrenia have reported fluctuations in ECP levels in affected individuals. Elevated serum or cerebrospinal fluid ECP levels have been observed in some cases, indicating a potential link between ECP dysregulation and the inflammatory processes characterizing schizophrenia. However, inconsistencies across studies necessitate further research to validate and standardize ECP as a reliable biomarker for schizophrenia.^[8,22]^

The observed alterations in ECP levels in schizophrenia patients suggest its potential involvement in the inflammatory cascade associated with the disorder. ECP’s presence in the peripheral circulation or CNS might reflect underlying immune dysregulation and neuroinflammatory mechanisms.^[23]^ Its correlation with the inflammatory milieu warrants exploration to determine its specific role and implications in schizophrenia pathophysiology. The potential utility of ECP as a biomarker in schizophrenia holds promise for disease monitoring and stratification.^[24]^ Monitoring ECP levels could provide insights into the dynamic immune alterations occurring during the course of the illness, potentially aiding in disease characterization and identifying subgroups with distinct immune profiles, allowing for personalized treatment approaches. Despite the intriguing findings, several challenges need to be addressed before considering ECP as a validated biomarker in schizophrenia. Variability in ECP measurements among studies, potential confounding factors, and the need for standardized assays and larger cohort studies represent critical aspects requiring attention.^[25,26]^

## 7. Neuroinflammatory implications of eosinophil cationic protein

The implications of ECP in neuroinflammatory processes, especially within the context of neuropsychiatric disorders like schizophrenia, remain an area of active investigation. While traditionally recognized for its roles in immune responses and host defense, recent research has suggested a potential link between ECP and neuroinflammation, shedding light on its possible involvement in CNS-related pathologies.^[27]^ Emerging evidence indicates that ECP, primarily released by activated eosinophils, might play a role in neuroinflammatory cascades within the CNS. Although the precise mechanisms remain unclear, the presence of ECP within the CNS or alterations in its levels in the cerebrospinal fluid (CSF) of individuals with neuropsychiatric conditions such as schizophrenia suggests its association with CNS inflammatory responses.^[28]^

ECP’s cytotoxic properties, including its ability to interact with cell membranes and induce cellular damage, raise questions about its potential impact on neural cells. It might influence neuronal function, synaptic plasticity, or neuronal viability within the CNS, potentially contributing to neuroinflammatory processes and affecting the delicate balance of CNS homeostasis in neuropsychiatric disorders.^[29]^ ECP’s release in the CNS might induce neurotoxic effects, triggering inflammatory cascades and exacerbating neuronal injury. Its interactions with glial cells or neurons could potentially promote the release of pro-inflammatory cytokines, such as IL-6 or TNF-α, amplifying neuroinflammatory responses and influencing disease progression in neuropsychiatric conditions.^[30]^ ECP’s potential role in modulating the BBB warrants attention. Dysregulation of ECP levels or its interactions within the BBB might compromise barrier integrity, facilitating the entry of inflammatory mediators or immune cells into the CNS. This disruption could contribute to neuroinflammation and impact neuronal function, potentially contributing to the pathophysiology of neuropsychiatric disorders.^[31]^ Understanding ECP’s involvement in neuroinflammatory mechanisms offers potential therapeutic implications. Strategies targeting ECP-related pathways or modulating its effects within the CNS might represent novel avenues for interventions aimed at mitigating neuroinflammation and ameliorating disease progression in neuropsychiatric conditions.

## 8. Therapeutic implications and future directions

The exploration of ECP and its potential involvement in neuroinflammation within neuropsychiatric disorders, including schizophrenia, offers promising avenues for therapeutic interventions and future research directions. Understanding the implications of ECP in CNS inflammatory processes presents opportunities for novel therapeutic strategies and advancements in the management of neuroinflammatory conditions.

## 9. Therapeutic implications

Developing therapies that specifically target ECP or its downstream effects could potentially modulate neuroinflammation. Strategies aimed at regulating ECP release, neutralizing its cytotoxic effects, or interfering with its interactions within the CNS might offer avenues for therapeutic interventions.^[32]^ Investigating compounds or interventions that dampen neuroinflammatory cascades triggered by ECP or its interactions might help mitigate neurotoxicity and neuronal damage in neuropsychiatric disorders. Strategies aimed at preserving BBB integrity and preventing dysregulation induced by ECP could be explored to limit the influx of inflammatory mediators into the CNS, potentially ameliorating neuroinflammation. Utilizing immunomodulatory agents targeting neuroinflammatory pathways influenced by ECP or immune dysregulation might offer therapeutic potential. These interventions could aim at restoring immune balance within the CNS.

## 10. Future directions and research focus

Further research is needed to delineate the precise mechanisms underlying ECP-mediated neuroinflammatory processes. Investigating ECP’s interactions with neural cells, its impact on neuronal function, and its contributions to neurotoxicity would provide crucial insights. Continuation of studies to validate ECP as a biomarker for neuropsychiatric disorders, including schizophrenia, is essential. Determining its specificity, sensitivity, and potential correlation with disease severity or treatment responses would enhance its utility.

Conducting well-designed clinical trials to evaluate therapies targeting ECP-related pathways or neuroinflammatory mechanisms is crucial. Investigating the safety and efficacy of potential interventions in patients with neuroinflammatory conditions could translate research findings into clinical applications. Utilizing preclinical models and translational approaches to study ECP’s effects in neuroinflammation will aid in understanding its role in disease pathogenesis and facilitate the development of targeted therapies. Exploring personalized treatment strategies based on individual immune profiles and ECP levels might offer tailored interventions for patients with neuroinflammatory disorders.

## 11. Conclusion

The exploration of ECP within the context of neuroinflammatory conditions, notably neuropsychiatric disorders like schizophrenia, presents a compelling area of research with significant therapeutic implications. ECP, traditionally recognized for its roles in immune responses and host defense, has emerged as a potential contributor to neuroinflammatory processes within the CNS. The evolving understanding of ECP’s involvement in CNS inflammatory cascades and its potential impact on neuronal function has sparked interest in its implications for neuropsychiatric conditions. Studies implicating altered ECP levels in schizophrenia suggest its association with the inflammatory milieu characterizing the disorder, opening avenues for considering ECP as a potential biomarker and therapeutic target.

The therapeutic implications stemming from understanding ECP-related neuroinflammatory pathways offer promising opportunities for developing novel interventions. Strategies targeting ECP-related mechanisms or modulating its effects on CNS inflammation present potential avenues for mitigating neurotoxicity and disease progression in neuropsychiatric disorders. While further research is warranted to clarify ECP’s role in neuroinflammatory processes, the exploration of ECP within neuropsychiatric disorders signifies a promising frontier in advancing our understanding and potentially transforming the management of these complex and challenging conditions.

## Author contributions

**Conceptualization:** Emmanuel Ifeanyi Obeagu.

**Methodology:** Emmanuel Ifeanyi Obeagu.

**Supervision:** Emmanuel Ifeanyi Obeagu.

**Visualization:** Emmanuel Ifeanyi Obeagu.

**Writing – original draft:** Emmanuel Ifeanyi Obeagu.

**Writing – review & editing:** Emmanuel Ifeanyi Obeagu.
